# Research Advances in Conjugated Polymer-Based Optical Sensor Arrays for Early Diagnosis of Clinical Diseases

**DOI:** 10.3390/polym18030310

**Published:** 2026-01-23

**Authors:** Qiuting Ye, Shijie Fan, Jieling Lao, Jiawei Xu, Xiyu Liu, Pan Wu

**Affiliations:** 1 College of Pharmacy, Guangxi Medical University, Nanning 530021, China; ye_qiuting@163.com (Q.Y.); 13807846373@163.com (S.F.); 2 State Key Laboratory of Targeting Oncology, Guangxi Medical University, Nanning 530021, China; 13107379400@163.com (J.L.); 18741957772@163.com (J.X.)

**Keywords:** conjugated polymers, sensor arrays, disease diagnosis, material design, biomedical applications

## Abstract

Early and accurate diagnosis is critical for disease surveillance, therapeutic guidance, and relapse monitoring. Sensor arrays have emerged as a multi-analyte detection tool via non-specific interactions to generate unique fingerprint patterns with high levels of selectivity and discrimination. Conjugated polymers (CPs), with their tunable π-conjugated backbones, exceptional light-harvesting capability, and efficient “molecular wire effect,” provide an ideal and versatile material platform for such arrays, enabling significant optical signal amplification and high sensitivity. This review systematically outlines the rational design and functionalization strategies of CPs for constructing high-performance sensor arrays. It delves into the structure–property relationships that govern their sensing performance, covering main-chain engineering, side-chain functionalization, and microenvironmental regulation. Representative applications are discussed, including non-small cell lung cancer, breast cancer, bacterial and viral infections, Alzheimer’s disease, and diabetic nephropathy, highlighting the remarkable diagnostic capabilities achieved through tailored CP materials. Finally, future perspectives are focused on novel material designs and device integration to advance this vibrant field.

## 1. Introduction

Early and accurate diagnosis of complex diseases demands analytical platforms that enable multiplexed and sensitive detection of biomarkers in clinical samples. In this context, sensor arrays have emerged as a powerful strategy, capable of generating distinct composite response patterns through the integration of multiple sensing elements [1,2,3]. The performance of such arrays is fundamentally governed by the properties of the sensing materials employed. Recent progress has incorporated various functional nanomaterials, including gold nanoparticles [4], carbon-based structures [5], and metal oxides [6]. Among these, conjugated polymers (CPs) stand out as a uniquely versatile class of polymeric sensing materials. Characterized by their π-conjugated backbones, CPs offer a synergistic combination of tailorable optoelectronic properties, synthetic versatility, and intrinsic signal amplification, establishing them as an ideal material platform for constructing next-generation sensor arrays [7,8,9].

CPs exhibit a broad spectral absorption range and a high molar extinction coefficient, which facilitate efficient exciton migration along the conjugated backbone and directed energy transfer to acceptor molecules. These characteristics underlie a pronounced signal amplification mechanism, often described as the “molecular wire” effect, leading to significantly lowered limits of detection (LOD) compared to conventional molecular probes [10,11]. Beyond this inherent advantage, the synthetic versatility of CPs represents a critical strength. Through rational molecular design, their optical profiles, solubility, and molecular recognition functionalities can be precisely engineered. This tunability enables the creation of customized sensing elements, making CPs exceptionally suitable for fabricating sensor arrays that produce discriminative response patterns, or “fingerprints,” for complex analytes. Owing to these distinctive photophysical attributes, CP-based sensing systems have been rapidly developed and successfully deployed in fields such as environmental monitoring and food safety [12,13,14,15,16,17], demonstrating reliable performance even within complex matrices.

Building on this foundation, integrating CPs into sensor arrays creates powerful synergies specifically tailored for clinical diagnostics. The inherent signal amplification of CPs, combined with the multiplexing capability of sensor arrays, allows for simultaneous and sensitive profiling of multiple disease biomarkers [18,19,20,21]. This capability addresses a pressing need in modern diagnostics, particularly for complex, multifactorial conditions such as cancer. Translating this potential into clinical practice requires a thorough understanding of the structure–property–application relationships in CPs. Physiological samples introduce matrix effects that can compromise analytical accuracy [22], underscoring the necessity for deliberate material design. Strategic engineering of the polymer backbone, side chains, and micro-environmental interactions is essential to enhance selectivity, stability, and real-world performance.

**Figure 1:**
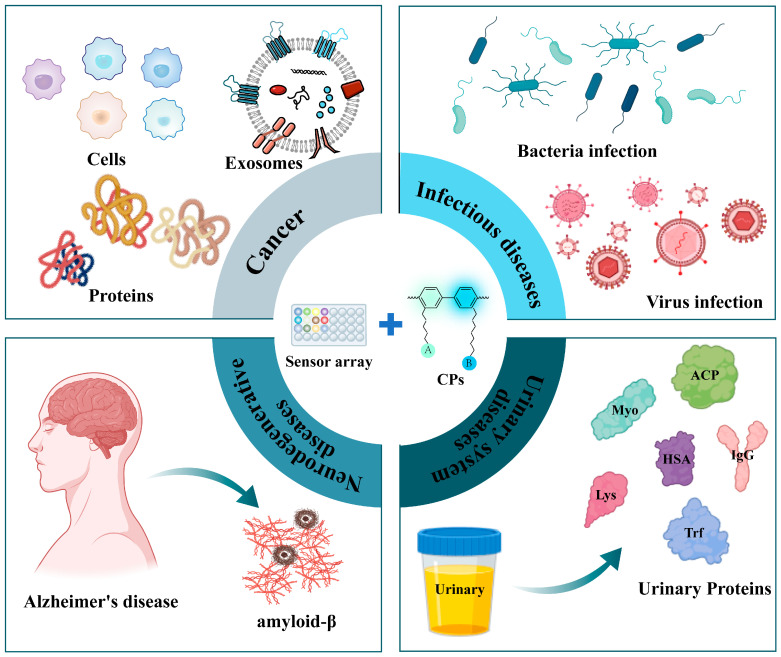
Schematic illustration of the applications of CP-based optical sensor arrays in early-stage clinical disease diagnosis. Created in BioRender. Q.Y. (2026) https://BioRender.com/3e2qkny.

This review provides a materials-centric perspective on the design and application of conjugated polymer-based sensor arrays for early disease diagnosis, with a specific focus on optical sensing platforms (Figure 1). In contrast to existing reviews that predominantly focus on the photophysical properties of conjugated polymers, single-analyte sensing systems, or broad biomedical applications [23,24,25], this work emphasizes the rational design strategies that bridge molecular structure to diagnostic function. Notably, recent reviews such as that by Phung and Tran discuss developments in CP-based fluorescent sensors for environmental and biological detection, highlighting their distinctive optical features and the molecular wire effect [8]. While these contributions aptly summarize advances in sensor performance and material properties, the present review offers a deeper examination of key material design strategies that are critical for optimizing sensor arrays in clinical practice, such as backbone engineering, side-chain functionalization, and microenvironmental regulation. We begin by exploring the fundamental material characteristics and signal amplification mechanisms of CPs, followed by a detailed discussion of key material design strategies for the construction of highly effective sensor arrays. Representative applications across cancer, infectious diseases, neurodegenerative disorders, and urinary system diseases are presented to illustrate how tailored material designs enable advanced sensing performance. By establishing a clear link between molecular-level engineering and functional outcomes, this review seeks to motivate continued innovation in the development of functional conjugated polymers for next-generation healthcare solutions.

## 2. Design and Construction of CPs-Based Optical Sensor Arrays

The design and construction of CPs-based sensor arrays involve the strategic synthesis and engineering of CP materials to optimize their sensing capabilities. CPs are characterized by a conjugated backbone that allows for efficient charge transport and signal amplification, making them ideal candidates for sensor applications. Several synthetic methods, such as electrochemical polymerization, oxidative polymerization, and transition-metal-catalyzed cross-coupling reactions, are employed to construct CPs with specific structural features that enhance their performance in sensor arrays. These CP-based arrays can generate unique response patterns by manipulating the polymer backbone, functional side chains, and microenvironmental factors, enabling precise and multiplexed detection of biomolecules or environmental pollutants.

### 2.1. Categories and Synthesis Methods of CPs

**Figure 2:**
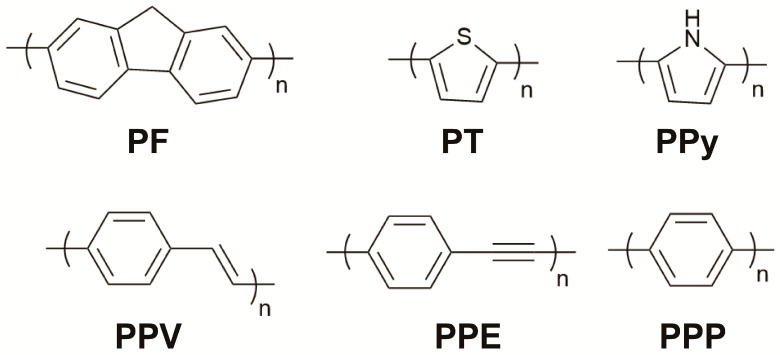
Chemical structures of some common CP backbones in sensor arrays.

CPs are characterized by a continuous π-electron delocalization system along their backbone, which provides exceptional light-harvesting capacity and strong fluorescence emission. This intrinsic optoelectronic property, coupled with synthetic versatility, forms the foundation for their sensing applications [26,27]. In practical applications of sensor arrays, common CPs include polyfluorene (PF) [28], polythiophene (PT) [29], polypyrrole (PPy) [30,31], poly(p-phenylene vinylene) (PPV) [32], and poly(p-phenylene ethynylene) (PPE) [33], poly(p-phenylene) (PPP) [34,35], etc. (Figure 2). From a synthetic perspective, CPs can be efficiently constructed through transition-metal-catalyzed cross-coupling reactions [36,37]. For instance, PPE-type polymers are typically synthesized via the Sonogashira coupling reaction, forming C–C bonds between alkynes and halogenated aromatic compounds under palladium catalysis. The Suzuki–Miyaura cross-coupling reaction is widely used for PF synthesis due to its excellent functional group tolerance and mild reaction conditions, allowing precise control over aromatic backbone formation and molecular weight distribution. PPy is synthesized through either chemical or electrochemical polymerization, where pyrrole monomers are oxidized by agents such as ferric chloride or by the application of a potential, resulting in conductive polymers. PT is commonly prepared via Stille coupling or FeCl_3_-mediated oxidative polymerization, whereas PPV is predominantly synthesized through the Heck reaction. PPP is synthesized through a cathodic-dehalogenation polymerization method. This high degree of synthetic control is paramount for materials design, enabling precise manipulation of the conjugated backbone, strategic introduction of functional side chains, and fine-tuning of molecular weight. These parameters directly and decisively influence the polymer’s optical properties, solution processability, and ultimately, its performance within a sensor array.

### 2.2. Fundamental Principles of CPs-Based Sensing

The main chain of CPs, formed by alternating single and double bonds, creates a π-π conjugated system that results in unique delocalized electronic properties [38]. Quantum chemical theory indicates that the presence of conjugated π bonds enables electrons within the molecule to delocalize along the entire backbone, significantly reducing the energy gap between the highest occupied molecular orbital (HOMO) and the lowest unoccupied molecular orbital (LUMO), thereby broadening the light absorption range. This structural feature synergizes with the intramolecular charge transfer effect, granting CPs strong light-harvesting capabilities, high fluorescence quantum yield, and excellent photostability [39].

**Figure 3:**
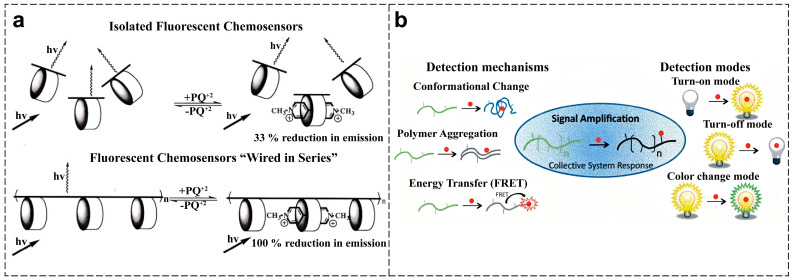
(**a**) Molecular wire effect [40]. (**b**) Detection mechanisms and modes for CP biosensors [44].

This unique structure enables CPs to exhibit a pronounced signal amplification effect. In 1995, Swager et al. first validated this phenomenon in a PPE system containing cyclopentadienyl receptors and named it the “molecular wire effect” (Figure 3a) [40]. The π-conjugated backbone acts as a one-dimensional channel for exciton migration. When an analyte binds to a receptor site on the polymer, the resulting local perturbation is communicated along the entire conjugated pathway through efficient energy or electron transfer. This leads to a cooperative optical response from numerous chromophores, dramatically amplifying the output signal. In biosensing applications, the linear molecular structure of CPs further strengthens this signal amplification mechanism. When the target analyte locally binds to specific recognition groups on CPs’ repeating units, intermolecular interactions efficiently propagate along the entire polymer backbone via the conjugated π-electron system. This induces molecular conformational changes and rearrangement of electron cloud distribution, resulting in significant modifications to the optical properties of the entire polymer chain. This synergistic response mechanism based on the conjugated system enables a single analyte-receptor binding event to trigger a collective response of chromophores along the entire conjugated chain, achieving signal amplification or quenching of fluorescence [41,42]. In contrast, small-molecule probes, lacking a long conjugated backbone, can only generate localized responses and exhibit relatively minor fluorescence quenching effects [43]. Therefore, CPs’ sensing systems, leveraging their signal amplification properties, exhibit significantly higher detection sensitivity for target analytes compared to small-molecule probes.

The signal amplification effect is closely related to the diverse photophysical response mechanisms of CPs. Its optical output typically manifests as “turn off” (quenching) or “turn on” (dequenching) of fluorescence intensity, changes in fluorescence color, and colorimetric detection (Figure 3b) [44]. In fluorescence quenching mechanisms, the optical property changes in CPs primarily result from photoinduced electron transfer (PET) [45], internal filtering effect (IFE) [46], Förster resonance energy transfer (FRET) [47], and aggregation-induced quenching (ACQ) caused by enhanced π–π stacking interactions between polymer chains [48]. Both PET and FRET rely on the distance between the probe and analyte, representing processes of electron and energy transfer. PET effectively quenches the fluorescence of CPs, enabling the construction of “on” or “off” type fluorescent probes based on this property. FRET, characterized by receptor emission quenching and donor emission enhancement, is suitable for constructing ratio-fluorescence probes, offering a versatile strategy for designing CP sensors with donor–acceptor structures. Unlike PET and FRET, IFE is fundamentally a reabsorption process whose mechanism does not rely on direct interaction between the analyte and the sensing material, leading to a faster response. Upon addition of the target analyte, the aforementioned quenching mechanisms regulate the fluorescence signal by altering the electronic structure or energy transfer pathways of the CPs. During de-quenching, analyte binding can restore fluorescence emission by disrupting the polymer backbone electron density or inducing chain conformation changes [9]. However, compared to quenching processes, the sensitivity of fluorescence dequenching systems is often lower. This is because some quencher molecules form strong quenching sites within the exciton diffusion length range, establishing stable interactions with the π-conjugated system of the CPs’ main chain through charge transfer or energy transfer mechanisms. The irreversible nature of this strong interaction-induced fluorescence quenching prevents the initial fluorescence signal from being fully restored during the dequenching phase. In fluorescence sensing systems, alterations in the electron density of the conjugated CPs backbone can induce changes in fluorescent color. Colorimetric sensing systems, however, offer an intuitive visual detection method, enabling direct visual assessment through changes in solution color. Colorimetric detection is typically triggered when the conjugated length of the CPs backbone changes due to recognition events, thereby modifying its light absorption wavelength [40]. The ability to harness and combine these mechanisms through material design is central to creating CPs with rich and analyzable optical outputs.

### 2.3. Strategies for Constructing CPs-Based Optical Sensor Arrays

**Figure 4:**
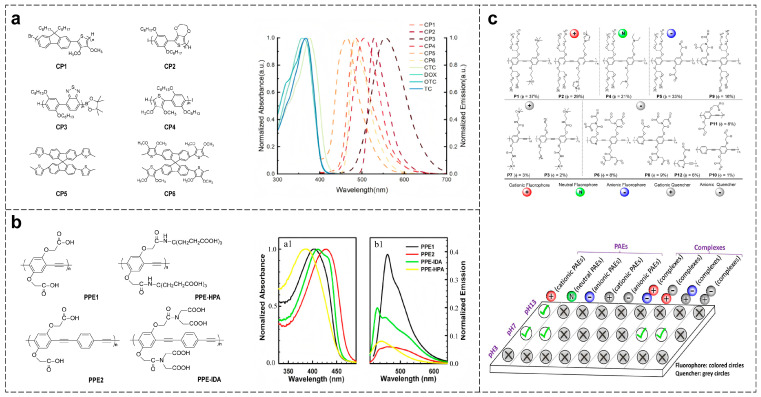
Optimizing CPs-based sensor arrays via structural and microenvironmental modulation. (**a**) Tuning optical properties through backbone engineering [49]. (**b**) Granting processability and recognition via side-chain modification, accompanied by the normalized (**a1**) UV-Vis absorption and (**b1**) fluorescence emission spectra of PPE derivatives in aqueous solution [50]. (**c**) Diversifying response patterns by microenvironmental regulation [51].

Performance optimization of CPs-based sensor arrays can be achieved through multi-level structural design and microenvironmental regulation. The core approach involves constructing a sensing system with differentiated response characteristics by engineering the main chain, functionalizing side chains, and modulating external conditions (Table 1). The backbone structure determines the optical properties of CPs, such as fluorescence quantum yield and the positions of absorption or emission peaks [49] (Figure 4a). Side-chain modifications confer solution processability and target recognition capabilities [50] (Figure 4b), while microenvironmental regulation further diversifies response patterns [51] (Figure 4c).

#### 2.3.1. Functionalization Strategy Based on Main Chain Modification

Regulating the main chain structure is a core strategy for modulating the optical properties of conjugated polymers. By introducing different conjugated units or performing structural modifications, the fluorescence characteristics of polymers can be systematically altered, laying the foundation for constructing sensing platforms with cross-response capabilities. Schanze et al. introduced heterocyclic units such as thiophene (Th) and pyridine (Py) into the poly(arylene ethynylene) (PAE) main chain. By leveraging donor–acceptor synergistic effects to modulate the HOMO–LUMO energy gap, they achieved a continuous red shift in fluorescence emission from blue to orange-red light, establishing a luminescent system spanning the visible spectrum [52].

**Table 1:** Design strategies of CPs -based sensor arrays for different analytical applications.

Functionalization Strategy	Class of Sensor Molecule	Mechanism	Analytes	Ref
Main Chain Modification	Poly(phenylene vinylidene), fluorene-thiazole copolymers, fluorene-anthracene copolymers	Electron transfer	Explosives	[53]
	Furan–thiophene, benzothiadiazole–benzene, spirobifluorene–thiophene backbones	FRET	Antibiotics	[49]
	PPETE, PPE	Chelation, electrostatic interactions, photoinduced electron transfer	Metal ions	[54]
	Acetylene groups, thiophene, bithiophene groups	IFE	Azo dyes	[55]
Side-Chain Modification	Iminodiacetic acid, hexylpropionic acid ester side chains	Aggregation, metal coordination	Metal ions	[50]
	Acid-containing, nitrogen-containing side chains	Charge distribution, hydrophilicity/hydrophobicity	Milk powder	[56]
	Carboxylate side chains	Electrostatic and hydrogen bonding interactions	Biogenic amines	[57]
Microenvironment Regulation	PPE1, PPE2, PPE1 + PPE2	pH-dependent electrostatic binding	Wine varieties	[58]
	PAE	pH-modulated charge state and complex stability	Aromatic carboxylic acids	[51]
	PTPEs, PPEs	Hydrophobic, electrostatic interactions	Flavonoid	[59]
	PPE1/metal ion complexes + GFP-K72	Synergistic interaction	Amino acids	[60]

Multiple teams have further explored the application of main chain structural regulation in sensing detection. Polcha et al. synthesized polymers with diverse backbone structures, including poly(phenylene vinylidene), fluorene-thiazole copolymers, and fluorene-anthracene copolymers. By exploiting their differentiated electron transfer reactions with explosive molecules, they achieved the identification of multiple explosives through changes in fluorescent signals [53]. Hu et al. designed CPs with furan–thiophene, benzothiadiazole–benzene, and spirobifluorene–thiophene backbones. Differences in spectral overlap between these polymers and tetracycline antibiotics led to varying FRET efficiencies, enabling highly efficient recognition of multiple antibiotics [49]. Xu et al. synthesized poly[p-(phenylethynyl)-alt-(thienylethynyl)] (PPETE) and PPE polymers. Through differential chelation, electrostatic interactions, and photoinduced electron transfer with metal cations, they achieved group-specific recognition of metal ions [54]. Jason D. Azoulay et al. designed copolymers with main chains containing acetylene groups, thiophene, and bithiophene groups. By modulating the polymer’s absorption or emission spectra, charge distribution, and spectral overlap with azo dyes through chain-specific variations, they employed IFE-induced fluorescence quenching to achieve efficient discrimination among 12 azo dyes [55]. These studies demonstrate that precise design of the main-chain structure provides a key pathway for constructing highly selective, broadly responsive, and differentiated sensing platforms.

#### 2.3.2. Functionalization Strategy Based on Side-Chain Modification

By introducing polar side chains or charged groups onto the polymer backbone, changes in the polymer framework phase can be induced, leading to the formation of ordered aggregated states. Side chain groups participate in analyte recognition through intermolecular forces such as electrostatic and hydrophobic interactions. The analyte-induced aggregation behavior promotes the formation of multi-recombinant assemblies with the polymer, triggering characteristic optical signal changes and generating a “fingerprint” response signal based on the structural features of the analyte.

Tan et al. designed CPs with identical PPE backbones but side chains functionalized with groups such as iminodiacetic acid and hexylpropionic acid ester. By controlling conjugation length, aggregation behavior, and metal coordination ability, combined with linear discrimination analysis (LDA), they achieved efficient discrimination of eight metal ions [50]. The team further developed CPs with sulfonic acid-containing (PPE-SO_3_) and nitrogen-containing (PPE-N1) side chains. By regulating charge distribution, hydrophilicity/hydrophobicity, and interactions with milk powder components through side chain structural variations, they achieved accurate classification of milk powders across different species, origins, and brands [56].

The multivalent nature of CPs makes them uniquely suited for detecting macromolecules with large recognition surfaces, such as proteins, cell surfaces, and other biomolecules. Lavigne et al. developed an array of CPs with carboxyl groups on the side chains. By exploiting differences in electrostatic and hydrogen bonding interactions between biogenic amines and polymers, they induced conformational changes in the main chain, generating unique optical fingerprints that enabled effective discrimination of seven biogenic amines [57]. This strategy fully demonstrates the pivotal role of side-chain modifications in regulating the analyte recognition capabilities of CPs, providing a universal approach for “fingerprint” detection of trace substances in complex systems.

#### 2.3.3. Construction of Response Patterns Based on Microenvironment Regulation

Microenvironmental regulation significantly expands the response dimensions and recognition capabilities of sensor arrays by modulating external conditions such as pH, solvent composition, and additives, which collectively tune the charge state, aggregation behavior, and analyte interactions of CPs. Among these factors, pH plays a critical regulatory role in determining the charge state of CPs and the stability of their complexes. Moreover, the behavior of numerous analytes in biological and natural systems undergoes significant changes in response to pH variations.

Bunz et al. systematically explored the role of pH in modulating the sensing performance of an array comprising anionic poly(p-phenylene ethynylene) (PPE1), its cationic counterpart (PPE2), and their electrostatic complexes. The distinct charge characteristics of these polymers are critical in governing their interactions with analytes and their responsive behavior across varying pH conditions. The study demonstrated that while PPE1 and PPE2 exhibited optimal responses at pH 13, their electrostatic complex produced effective signal outputs at both pH 3 and 13. This system binds to charged components in wine via electrostatic interactions, triggering fluorescence quenching and generating distinct fluorescence response patterns. Combined with LDA, this pH-regulated strategy successfully achieved accurate identification of 13 wine varieties [58]. In the detection of aromatic carboxylic acids, the team achieved efficient differentiation of 21 aromatic carboxylic acids by modulating the charge state and complex stability of ion-type PAE through pH adjustments at pH 7 and pH 13 [51]. In flavonoid identification, six-dimensional response fingerprints were constructed using five distinct fluorescent CPs under acidic and alkaline conditions. The CP panel comprised two poly(tetraphenylethene)s (PTPEs), along with three water-soluble poly(para-phenyleneethynylene) s (PPEs). The variation in pH modulates the hydrophobic and electrostatic interactions between the CPs and flavonoids, leading to differential fluorescence responses that provide selectivity among structurally similar analytes. This system successfully discriminated 11 flavonoids and related commercial products [59].

Introducing functional additives alongside solvent regulation can further enhance array performance. Bunz et al. constructed a positively charged PPE1 that forms complexes with metal ions at three pH values. When amino acids coordinate with metal ions, the complex structure is disrupted, triggering changes in the PPE1 fluorescence signal. Classification was achieved via LDA, but the single PPE system demonstrated only 77.5% recognition accuracy for unknown amino acids. By integrating green fluorescent protein (GFP-K72) into the PPE/metal ion system to form a new array, the synergistic interaction with metal ions enhanced signal diversity and improved recognition accuracy in complex systems. This increased the accuracy of identifying unknown samples from 77.5% to 86.3% [60].

## 3. Data Analysis and Pattern Recognition Methods

The sophisticated optical signals generated by CP-based sensor arrays yield high-dimensional data, necessitating advanced analytical methods to extract meaningful diagnostic information. The effective interpretation of these complex “fingerprint” responses is crucial for validating the performance of the sensing materials and realizing the full potential of the array platform. Machine learning algorithms have thus become integral to this process, offering powerful tools for pattern recognition and classification [61]. These algorithms are essential for deciphering the multivariate response patterns produced by CP arrays, effectively bridging the gap between raw optical output and actionable diagnostic readouts [1,62].

### 3.1. Unsupervised Learning Algorithms

Unsupervised learning techniques offer powerful, label-free methods for the initial interrogation of sensor array data. Their primary value in a clinical materials context lies in visually elucidating whether the engineered response patterns of a CP array can inherently distinguish between clinically relevant sample categories, such as healthy versus diseased states or different pathogen types. This provides an intuitive, preliminary validation of the material system’s discriminatory capability.

Principal Component Analysis (PCA) is extensively employed to reduce the dimensionality of complex fluorescence or colorimetric response data. By projecting high-dimensional data into a lower-dimensional space while preserving maximal variance, PCA produces readily interpretable score plots. These plots allow researchers to visually assess the clustering of samples, offering immediate insight into the array’s ability to separate distinct clinical cohorts based solely on their material interaction signatures [63,64]. In the context of CP arrays, PCA is frequently used to visualize the clustering of different sample types based on their composite optical fingerprints. For example, it has been employed to differentiate bacterial strains based on their distinct interaction patterns with CP sensors, clearly separating categories such as Gram-positive and Gram-negative bacteria or various antibiotic-resistant phenotypes, demonstrating the material’s potential for rapid antimicrobial resistance profiling [65].

Hierarchical Cluster Analysis (HCA) organizes samples into dendrograms based on the similarity of their response vectors. The branching structure and linkage distances provide a quantitative, hierarchical map of sample relatedness. This method is particularly useful for revealing subgroups or phylogenetic relationships within a data set [66]. For instance, HCA has been successfully applied to cluster different aggregation states of amyloid-β proteins based on their unique fluorescence response patterns to a CP-based array, demonstrating clear separation between monomers, oligomers, and fibrils [67].

### 3.2. Supervised Learning Algorithms

Supervised learning algorithms are used to construct predictive models that establish a rigorous, quantitative link between the array’s response and specific clinical outcomes (classification) or analyte concentrations (regression). This step is paramount for translating a material’s sensing capability into a reliable prognostic or diagnostic tool.

Linear Discriminant Analysis (LDA) is a robust and widely used method for classification tasks. LDA identifies a linear combination of sensor responses that maximizes the separation between predefined clinical classes. The resulting model provides a clear, simplified projection of the data where class discrimination is optimized, yielding a straightforward metric for diagnostic accuracy [68]. For example, LDA has been successfully applied to demonstrate the high classification accuracy of a tailored CP electronic tongue in discriminating between different tetracycline antibiotics, showcasing direct translation from material-analyte interaction to compound identification [49].

Artificial Neural Networks (ANNs) and Deep Learning represent advanced paradigms capable of modeling highly complex, non-linear relationships within data [69]. Their integration with CP sensor arrays embodies a “materials informatics” approach, where sophisticated computational models unlock deeper diagnostic insights from intricate material-response landscapes. ANNs excel at processing the nuanced patterns generated when arrays interact with complex biological matrices like serum or tissue lysates. Through training, these networks learn to associate subtle features in the optical response with specific clinical endpoints [70]. An exemplar is the integration of a multi-signal nanozyme sensor array with an ANN to achieve simultaneous quantification of multiple antioxidants in complex samples, highlighting the synergy between advanced nanocomposite design and intelligent data processing for multiplexed clinical analysis [71].

## 4. Application in Clinical Disease Diagnosis

The rational design and synthesis of CPs enable the construction of sensor arrays with enhanced sensitivity, specificity, and multiplexing capabilities, forming the foundation of their diagnostic potential. These material platforms generate quantifiable optical signals, primarily through fluorescence or colorimetric readouts, leveraging well-defined photophysical mechanisms. This tailored materials approach facilitates rapid and specific disease identification, supporting advancements in personalized medicine and point-of-care diagnostics.

### 4.1. Cancer Diagnosis

Malignant tumors represent a significant global health challenge, often requiring early and precise detection for effective intervention [72]. Current clinical diagnostic approaches include imaging examinations, pathological examinations, serological tests, and endoscopic examinations [73,74]. However, due to significant variations in baseline expression of target biomarkers across different populations and the lack of ideal biomarkers for most cancer types, false positive and false negative results frequently occur in practical applications. Sensor arrays, inspired by the olfactory and gustatory systems, offer a promising solution for more reliable cancer detection. The human olfactory system, for instance, uses an array of receptors to identify specific odors, while the gustatory system detects a variety of flavors through taste receptors. These biological systems are highly sensitive and selective. Biomimetic sensor arrays mimic this capability by employing a set of sensors with distinct chemical sensitivities, allowing for the detection and discrimination of multiple analytes. This “chemical olfaction” technology, driven by advanced material design, provides a powerful alternative for biomarker sensing with multiplexed detection.

#### 4.1.1. Cell Phenotype Analysis

**Figure 5:**
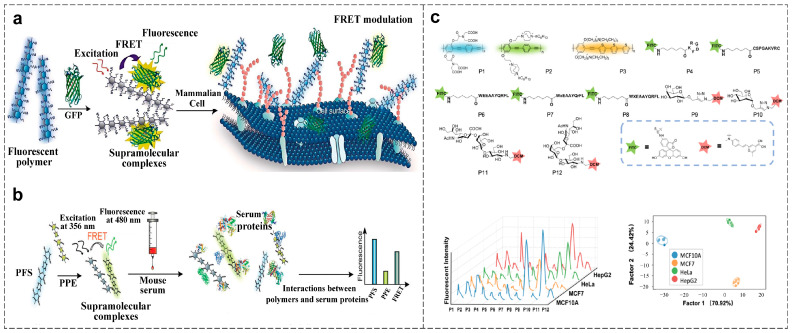
(**a**) Schematic diagram of the sensing array composed of CPs and GFP for cell phenotyping analysis [76]. (**b**) Schematic diagram of the sensing array composed of PFS and PPE derivatives for protein detection in serum [79]. (**c**) Schematic diagram of the sensor array composed of CPEs, fluorescently labeled peptides, and modified sugars for the detection of cells and exosomes [80].

Cell lysates reflect comprehensive phenotypic variations and serve as effective targets for cellular identification. This characteristic makes cellular phenotype analysis an increasingly valuable tool for cancer research and treatment [75]. Rotello et al. developed a high-precision sensing platform based on a FRET pair comprising cationic CPs and anionic green fluorescent protein (GFP) (Figure 5a). This design exploits electrostatic complex formation and subsequent cell-surface-induced disassembly. Different cell lines disrupt these preformed complexes in unique ways, generating distinct FRET modulation patterns. This approach illustrates how supramolecular interactions in CP hybrids can be strategically programmed to create powerful tools for discriminating between normal, cancerous, and metastatic cell lines with high accuracy [76].

#### 4.1.2. Biomarker Detection

Biomarkers, as disease-specific indicators, are widely used in clinical diagnosis [77]. In early cancer detection, accurate identification of specific proteins and biomarkers such as exosomes is crucial for prevention and treatment. Additionally, biomarkers located on the cell surface have become important detection targets due to their non-invasive acquisition [78].

Proteins, as fundamental components of cells and tissues, often exhibit expression and structural alterations linked to disease states. Thus, their efficient detection and differentiation are vital for clinical diagnosis. Rotello et al. developed a detection system based on FRET between conjugated polymers and competitive serum protein binding (Figure 5b). This design leverages the unique properties of CPs, particularly their signal amplification and spectral tunability, achieved by the engineered polymer backbone and side-chain functionalities. The system detects fluorescence ratio changes, which are primarily driven by molecular-level interactions between the CPs and proteins. By employing these engineering strategies, the system successfully distinguished eight protein types with 97.0% accuracy, demonstrating how material-driven signal amplification facilitates the detection of subtle biomarker shifts even in complex biological samples. The system achieved 89.1% accuracy for distinguishing healthy from cancerous serum samples, further validating the critical role of CPs in improving sensitivity in clinical diagnostics [79].

Exosomes, which carry various tumor-associated proteins inherited from parental cells, are regarded as potential biomarkers for early cancer diagnosis. The identification of their surface molecular characteristics serves as an effective approach for cancer diagnosis and classification. Tan et al. constructed a 12-unit fluorescent sensor array comprising three conjugated polyelectrolytes, five fluorophore-labeled peptides, and four cyanopyranosyl-modified sugars (Figure 5c). By engineering CPs with optimized photophysical properties and integrating them with biofunctional peptides and sugars, the array achieves multivalent interactions with exosome components, generating distinct fluorescence responses. The array achieved 100% classification accuracy for normal cells, cancer cells, and distinct breast cancer subtypes, demonstrating how conjugated polymer-based sensor arrays leverage material engineering to distinguish biomarker shifts. To address the complexity of plasma-derived exosomes, the team utilized the deep learning model AggMapNet to convert unordered fluorescence spectra into feature maps (Fmaps). By applying machine learning techniques to analyze polymer-generated fluorescence fingerprints, the model achieved 100% accuracy in predicting breast cancer patients versus healthy individuals based on these distinctive fluorescence patterns [80]. The integration of CP arrays with deep learning enhances the accuracy and sensitivity of tumor-derived exosome detection, presenting a promising strategy for early, minimally invasive cancer diagnosis.

### 4.2. Infectious Diseases Diagnosis

Infectious diseases are caused by pathogenic microorganisms such as bacteria and viruses that invade the human body through routes including the respiratory tract or bloodstream, leading to physiological damage. Current diagnostic methods for infections typically involve pathogen culture, serological testing, and molecular assays. However, these traditional techniques often face challenges of low detection efficiency and insufficient accuracy due to their reliance on specialized equipment and personnel, complex operations, lengthy testing cycles, and difficulty in addressing the significant differences in biological characteristics among various pathogens [81,82]. In contrast, array-based biosensing technology enables high-throughput, highly sensitive pathogen detection by integrating multiple sensing elements.

**Figure 6:**
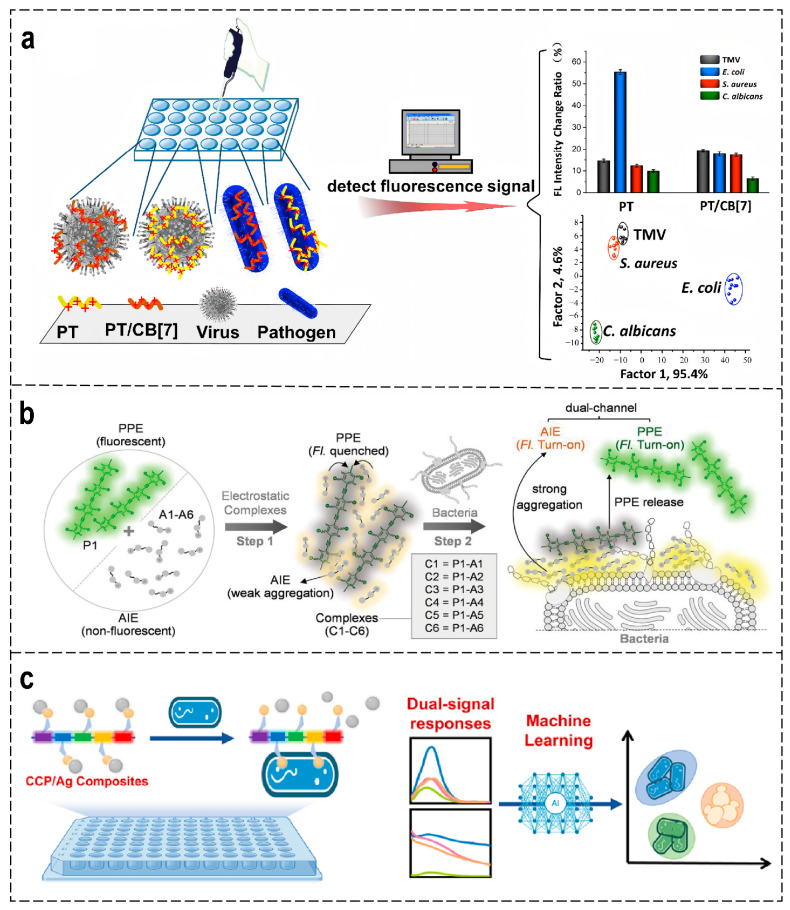
(**a**) Schematic diagram of the sensing array composed of PT derivatives and CB [7] for virus and microbes identification [83]. (**b**) Schematic diagram of the dual-fluorescence sensor array composed of PPE (P1) and AIE molecules (A1–A6) for bacterial identification [84]. (**c**) Schematic diagram of the dual-mode sensing array composed of CCP and AgNPs for bacteria and fungi discrimination [85].

Wang et al. developed a CPs-based sensor array using supramolecular complexes formed between cationic polythiophene derivatives and Cucurbit [7] uril (CB [7]) (Figure 6a). Through the distinct fluorescence intensity patterns generated by their interactions with different biological samples, LDA processing separates viruses and microbes into four independent clusters with 99.9% accuracy. This method requires no complex synthesis and enables rapid identification through fluorescence signals and pattern recognition, offering a novel strategy for rational antibiotic use and biosensing [83]. The distinct fluorescence fingerprints generated by this supramolecular CP array allow for the accurate and rapid identification of pathogens, suggesting its utility in fast clinical screening.

To enhance binding and optical response, hybrid material designs have been employed. Aggregation-induced emission (AIE) molecules are a class of polymorphic molecules with unique optical properties. In their free state, they do not emit light due to non-radiative energy dissipation caused by molecular backbone rotation. However, in their aggregated state, restricted molecular motion enables highly efficient fluorescence emission. By leveraging the ability to chemically modify polymer monomers to regulate bacterial binding capacity, AIE sensor arrays have emerged as a convenient tool for bacterial recognition. Han et al. constructed a dual-fluorescence turn-on sensor array based on electrostatic complexes between an anionic PPE (P1) and six cationic AIE molecules (A1–A6) (Figure 6b). Negatively charged bacterial surfaces disrupt these complexes, triggering dual-channel fluorescence changes that, combined with machine learning, enable bacterial identification. This array can identify 20 bacterial species within 30 s, maintaining high accuracy at both OD600 = 0.005 and 0.001, outperforming single-element arrays. In a blind test of 28 urine samples, the sensor array achieved a 96.4% identification accuracy, significantly outperforming quantitative polymerase chain reaction (qPCR). The random forest model demonstrated a prediction accuracy of 96.7%, with an area under the receiver operating characteristic curve (AUROC) of 0.99 [84]. Thus, CP/AIE hybrid sensor arrays can help identify bacterial species through rapid dual-channel fluorescence responses with high accuracy, which is promising for point-of-care infection diagnosis.

Silver nanoparticles (AgNPs) exhibit outstanding stability, excellent water solubility, high molar extinction coefficient, and unique localized surface plasmon resonance (LSPR) properties. Compared to gold nanoparticles, AgNPs possess a higher free electron density, demonstrating not only stronger LSPR responses but also significantly enhanced molar extinction coefficients. In combination with conjugated polymers, AgNPs improve both signal amplification and target specificity through synergistic electrostatic and optical interactions. Bai et al. developed a dual-mode fluorescence-colorimetric sensing array utilizing CCP/Ag composites, which were formed through electrostatic assembly of cationic conjugated polymers (CCP) and AgNPs (Figure 6c). This array leverages hydrophobic and electrostatic interactions with microorganisms, along with the LSPR effect of AgNPs, to generate dual optical responses comprising CCP fluorescence and CCP/Ag colorimetric signals upon binding to pathogens with diverse surface structures. This design enables simultaneous microbial detection and concentration quantification, facilitating rapid, high-throughput discrimination of Gram-negative bacteria, Gram-positive bacteria, and fungi. The system demonstrated 100% classification accuracy during validation and maintained robust performance in complex sample matrices [85]. By leveraging dual-mode optical signals, CCP/Ag nanocomposite arrays achieve highly accurate and high-throughput discrimination of microbial classes, holding promise for advanced microbiological detection.

### 4.3. Neurodegenerative Diseases Diagnosis

Neurodegenerative diseases, including Alzheimer’s disease (AD), Parkinson’s disease, Huntington’s disease, amyotrophic lateral sclerosis, and multiple sclerosis [86], are characterized by pathological processes closely associated with oxidative stress. Oxidative stress plays a critical role in the progression of these disorders, contributing to neuronal death and impaired neural function. Protein oxidation represents a common pathological feature, with amyloid-β (Aβ) particularly implicated in disrupting metal ion homeostasis within the brain [87,88]. Consequently, monitoring Aβ levels in biological fluids provides a valuable diagnostic and prognostic marker for neurodegenerative conditions. Current Aβ detection methodologies primarily encompass optical techniques, neuroimaging, and immunoassays [89,90]. However, these approaches are often constrained by high costs, time-consuming protocols, and limited suitability for high-throughput analysis. Therefore, the development of highly sensitive and accurate methods for early-stage Aβ detection represents an urgent need. In contrast, sensor arrays based on CPs offer distinct advantages for trace-level detection.

**Figure 7:**
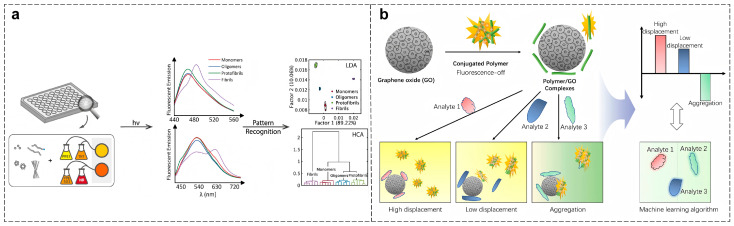
(**a**) Schematic diagram of the cross-reactive sensor array composed of PPE1, PPESO_3_, and organic dyes for distinguishing Aβ42 morphologies [67]. (**b**) Schematic diagram of the sensing platform composed of PPE and GO for identifying Aβ [91].

Tan et al. developed a cross-reactive fluorescent sensor array based on two pairs of probes (Figure 7a). The unique photophysical properties of CPs, particularly their high fluorescence quantum yield and sensitivity to conformational changes, allow for the detection of subtle alterations in protein aggregation states. The system generates multidimensional response signals, capturing changes in fluorescence intensity and spectral shifts, which enable the differentiation of Aβ42 aggregates into monomers, oligomers, protofibrils, and fibrils. The integration of CPs with dyes creates a robust platform for identifying different aggregation states, providing a tool with 100% accuracy, as demonstrated through leave-one-out cross-validation (LOOCV). Furthermore, the system was able to distinguish between “on-pathway” and “off-pathway” aggregation mechanisms, offering valuable insights into the kinetics of protein aggregation and its inhibition, which is crucial for understanding neurodegenerative disease progression [67]. This approach underscores the critical role of CPs as functional materials in generating accurate diagnostic signals for complex biological systems.

Graphene oxide (GO) has gained widespread application as a fluorescence quencher in sensing systems due to its excellent aqueous dispersibility, efficient multivalent quenching capability, and versatile surface functionalization properties. Han et al. developed a sensor array based on electrostatic complexes formed between fluorescent conjugated polymers and GO, combined with machine learning algorithms for identifying Aβ aggregate morphologies (Figure 7b). The combination of CPs and GO creates a highly sensitive system that relies on variations in fluorescence intensity resulting from charge and conformational differences among distinct Aβ aggregates. The polymer-derived fluorescence responses serve as unique “fingerprints” for Aβ, enabling machine learning models to classify aggregates based on their morphology. In experimental validation, the array achieved 100% accuracy in distinguishing Aβ40 and Aβ42 aggregates, with further optimization demonstrating 100% classification accuracy for 12 distinct proteins. By utilizing CPs as the core functional material, the array successfully identifies unknown samples in blind testing, highlighting the potential of CP-based sensing platforms in the early diagnosis of neurodegenerative diseases [91]. This integration of materials science and computational analysis represents a promising direction for high-accuracy, early-stage diagnostics in complex disease systems.

### 4.4. Urinary System Diseases Diagnosis

Urinary system diseases refer to various conditions that impair the normal function of the human urinary system [92]. Damage or dysfunction in the kidneys or urogenital tract can lead to proteinuria, which represents one of the key clinical manifestations of urinary system diseases. Alterations in the composition and concentration of urinary proteins are closely associated with various urinary system pathologies [93]. While conventional detection methods such as mass spectrometry and ELISA offer high sensitivity and selectivity, they are constrained by limitations including high cost and operational complexity, rendering them unsuitable for high-throughput screening requirements. As a result, the development of rapid, sensitive, and cost-effective protein detection platforms is essential to meet the growing demand for point-of-care diagnostics.

**Figure 8:**
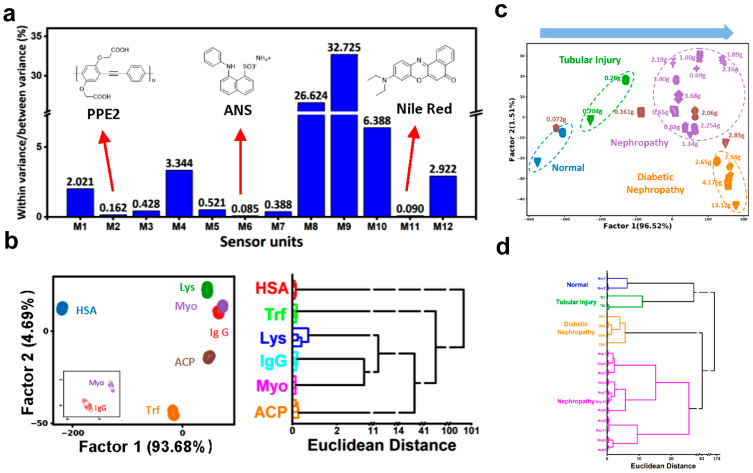
(**a**) Fluorescence response patterns of 12 fluorescent molecules for discriminating different proteins, and structures of the three fluorophores used in the optimized sensor array. (**b**) LDA score plot and HCA dendrogram for detecting six different proteins using the optimized three-unit sensor array. (**c**) LDA score plot and (**d**) HCA dendrogram for discriminating urine samples from patients with various urinary system diseases and healthy individuals [94].

Jiang et al. developed a three-unit sensor array incorporating fluorescent molecules ANS, NR, and PPE2 to detect urinary proteins (Figure 8a). The CPs in this array provide highly sensitive fluorescence responses, which are crucial for detecting minute changes in protein concentration. By leveraging the distinctive photophysical properties of CPs, this system achieves precise classification of urinary proteins through pattern recognition algorithms such as LDA and HCA (Figure 8b). The array demonstrated 100% accuracy in identifying six individual proteins and successfully distinguished between simulated protein mixtures in both PBS and healthy human urine samples. Clinical validation of the array further confirmed its ability to accurately differentiate urine samples from patients with tubular injury and diabetic nephropathy, although some overlap was observed with systemic lupus erythematosus (SLE) cases (Figure 8c,d). This sensor array requires no specialized instrumentation and enables rapid detection, thereby providing an effective strategy for non-invasive diagnosis of urinary system diseases [94]. Through pattern recognition of urinary protein fingerprints, small-molecule/CP hybrid sensor arrays offer a rapid, accurate, and instrument-free method for diagnosing urinary system diseases, aligning well with the needs of point-of-care testing.

## 5. Summary and Outlook

This review systematically summarizes recent advances in CP-based sensor arrays for early clinical disease diagnosis, focusing on their material properties, array construction strategies, and practical diagnostic applications. CPs exploit their π-conjugated backbones to achieve broad-spectrum light absorption, high molar extinction coefficients, and a molecular wire effect, which collectively enable efficient signal amplification and help overcome the limitations of conventional detection methods. By integrating multiple design strategies such as backbone modification, side-chain functionalization, and microenvironment control into cross-responsive sensor arrays and combining them with pattern recognition algorithms, including PCA and LDA, this platform allows for the simultaneous detection of multiple analytes within complex biological matrices, thereby enhancing diagnostic accuracy and reliability. As summarized in Table 2, CP-based arrays have demonstrated considerable promise across a spectrum of clinical conditions, including cancer, infectious diseases, neurological disorders, and urinary system diseases.

Nevertheless, several critical challenges must be addressed before these sensor arrays can achieve widespread clinical adoption. One of the primary hurdles is the nonspecific interference from complex biological matrices, which can lead to inaccurate sensor readings due to the presence of numerous coexisting biomolecules that may affect the sensitivity and specificity of the detection. Overcoming this issue will require the development of more refined sensor designs and advanced signal processing techniques to isolate relevant signals from background noise. Another significant challenge lies in the scalable synthesis of functional polymers. While small-scale synthesis has shown promising results, the ability to produce these materials on a larger scale, with consistent quality and functionality, remains a limitation that hampers the widespread use of CP-based sensor arrays in clinical settings. Finally, thorough biocompatibility validation is essential for ensuring the safety and long-term stability of these sensors when used in human diagnostics. Large-scale clinical studies are necessary to assess their reliability, potential toxicity, and overall performance in real-world medical environments.

Despite these challenges, the future of CP-based sensor arrays in disease diagnosis and monitoring remains promising. Advances in polymer chemistry, nanotechnology, and machine learning have provided a solid foundation for overcoming these obstacles. For instance, the development of more robust and selective sensor materials, combined with improved data analysis techniques, will likely enhance the reliability and precision of these arrays. Furthermore, the integration of artificial intelligence in processing complex datasets could significantly improve the detection capabilities and clinical utility of CP-based systems. As these challenges are addressed, CP-based sensor arrays are poised to become a valuable tool for early disease detection and personalized medicine, offering rapid, cost-effective, and non-invasive diagnostic solutions for a wide range of medical conditions.

## Figures and Tables

**Table 2:** Overview of CPs-Based Sensor Arrays for Early Diagnosis of Clinical Diseases.

Clinic Disease	Class of Sensor Molecule	Analytes	Analysis Method	Ref
Cancer	PPE/GFP	cells	LDA, HCA	[76]
	PPE, PFS	serum protein	LDA	[79]
	PPE	cells, exosomes	LDA, Deep learning	[80]
Infectious Diseases	PT/CB [7]	virus, microbes	LDA	[83]
	PPE/AIE	bacteria	LDA, Deep learning	[84]
	CCP/Ag	bacteria, fungi	LDA, PCA	[85]
Neurodegenerative diseases	PPE, ThT, NR	Aβ	LDA, HCA	[67]
	PPE/GO	Aβ	LDA, Deep learning	[91]
Urinary System Diseases	PPE, ANS, NR	urine proteins	LDA, HCA	[94]

## Data Availability

No new data were created or analyzed in this study.
